# Investigating mass balance of Parvati glacier in Himalaya using satellite imagery based model

**DOI:** 10.1038/s41598-020-69203-8

**Published:** 2020-07-22

**Authors:** Swati Tak, Ashok K. Keshari

**Affiliations:** Department of Civil Engineering, Indian Institute of Technology, Delhi, New Delhi 110016 India

**Keywords:** Cryospheric science, Environmental impact, Hydrology, Cryospheric science

## Abstract

Accurate assessment of glacier mass loss is essential for understanding the glacier sensitivity to climate change and the ramifications of glacier retreat or surge. The glacier melt affects the runoff and water availability, on which the drinking and irrigation water supplies and generation of hydroelectric energy depend upon. The excessive glacial retreat may cause flood, glacial lake outburst flood, avalanches and sea level rise which are likely to affect the lives and livelihood of the people and damage the infrastructure. Here, we present a remote sensing based modeling framework to improve the understanding of accumulation and ablation processes and to quantify the glacier mass balance using multispectral satellite imageries, as several glacierized regions of the world are still poorly monitored because the field measurements for continuous monitoring on a large scale or in a complex harsh terrain are costly, time consuming and difficult. The developed modeling framework has been applied to the Parvati glacier in the western Himalaya to investigate glaciological processes and estimate the surface mass loss using 19 years of satellite images from 1998 to 2016. It spreads over 425.318 km^2^ and more than 50% of the area is accumulation area. The study shows that the Parvati glacier is not in equilibrium and its behavioural response changes year to year characterized with high rate of mass loss. The value of accumulation area ratio varies between 0.33 and 0.70 with an average value of 0.55, indicating a negative mass loss. The mean specific mass loss is − 0.49 ± 0.11 m w.e. and the total mass loss is 3.95 Gt., indicating strong influence of climate change and effect on river flows and water availability.

## Introduction

There has been increasing worldwide interest in glacier studies as glaciers are considered among the most sensitive indicators of climate change because they are one among many natural entities that are directly affected by the climate change, and several model studies show that the climate change due to global warming will result in significant melting of earth’s glaciers and thereby a significant rise in global sea level. It is evident from literature review that there are several regions with highly negative mass balances, but there are also regions with positive mass balances and some regions in equilibrium condition^1–5^. For example, the glaciers that are showing negative mass balances and therefore shrinking with time include alpine glaciers in Europe^1^ and Himalayan glaciers in India^2–4^. The glaciers that are growing with time and showing positive mass balances are Scandinavian glaciers^1^. However, the mass balances of glaciers in the Caucasus^5^ are close to zero indicating that they are in equilibrium, not showing any signature of growing or shrinking with time. A regional glacial mass balance potential was obtained for 300 years to study glacial dynamics in the Glacial National Park, Montana and the obtained results were compared with the historic values of advances and retreats of Jackson and Agassiz glaciers in the northwest Montana^6^. This study utilized tree-ring based reconstructions of surface temperature anomalies in North Pacific and summer drought as representative indicators for the winter glacial accumulation and the summer ablation, respectively. The earlier study for these two glaciers had indicated a moderate retreat of about 3–14 m/year till 1917, and then after a very high retreat of greater than 100 m/year for about 25 years, indicating a remarkable shift of the glacial mass balance potential to an extremely negative phase^7^. The recession in the Swiss Alps glacier was examined for several thousand years based on the radiocarbon derived ages of materials that were found in the proglacial fluvial sediments of the subglacial origin^8^. These materials primarily include subsoil remains of wood and peat. Based on the analysis, a master chronology of fluctuations of Swiss glacier was constructed for the period of the Holocene (1,200–9,850 cal. yr BP or BC 7,900–AD 750). The ice and meteorological measurements made during 1865–2006 were analyzed to compute the annual mass balances of a number of Swiss Alps glaciers, namely, Silvretta, Rhone, Aletsch and Gries^9^. It was observed that these glaciers have decreased in size, but the rate of glacier shrinkage has not accelerated with time. A number of recent studies on glaciers reveal that most of the glaciers will disappear by a time less than a decade if the present trend of retreat remains^10^. A comparative analysis of retreat rates of Alaskan glaciers shows that the retreat rates are faster after 1971 and are still continuing^11^.

In-situ measurements of mass balance of a glacier provide not only strong evidence for the glacier retreat and climatic sensitivity, but also provide a benchmark data set for the verification and reliability of results of mass balances estimated from other methods that may be empirical, statistical, mathematical, hydrological, or remote sensing technique. However, the great challenge remains in deciding the number of in-situ measurements and at what locations it should be performed. One of the study^12^ on this aspect dealt with the appropriate number of stakes required to be installed for the measurement of mass balance of a glacier, and it shows that the gradient of the mass balance with altitude is more dominant than the transverse variations in deciding the number of measurements. Later, another study^13^ thoroughly investigated this aspect by considering a dynamical system of in-situ mass balance measurements wherein a second order spatial field model was presented for the statistical optimality of stakes to arrive at the number and locations of stakes distributed over the areal extent of the glacier for the measurement of mass balance in the field. This study also evaluated the efficiencies of monotonic and space-filling designs with the real data sets for the Chilean glaciers, namely, Olivares Alfa and Beta that contribute significantly to the stream flow of the Maipo river, and observed that the variance can increase or decrease due to the removal of some stakes under a specified covariance structure. It also advocated that the statistical designs are important where the simple application of raster design leads to increase in variance. The dynamical systems having generic conditions underline the empirically observed fact that around 11 well-positioned stakes are sufficient for mass balance measurement in homogenous glaciers.

The literature review shows that the studies on the Himalayan glaciers are very limited. These studies include geomorphology, mass balance and dynamics of glaciers, and are mainly based on the field measurements of snow and meteorological parameters, and remote sensing data consisting of aerial photographs and satellite images. It may be noted while using remote sensing products that the glacier movements can be calculated only after the removal of topographic information, otherwise the images will be replete with noise due to geometry. The estimation of glacier mass loss using parameters is possible only when the precise ortho-rectification of nonmoving objects is done in images that can be identified using fixed points. A number of geomorphological parameters including glacier shape and size, unique glaciological features, hypsometry, and bed topography can be investigated using remote sensing based techniques, and these parameters and features are very helpful in understanding the glacier mass balance. The presence of supra-glacial lakes in the region points to subsidence and fast degenerating nature of the glacierized area. However, some studies^14^ use numerical model with climatic datasets due to limited field data on glacier, rather than making use of remote sensing data, for understanding the glacier retreat. The numerical ice flow model^14^ was developed for a glacier in the Nepal Himalaya to investigate the role of local climate on the glacier retreat and the evolution of glacier in future. The topographical characteristics of Hamtah and Chhota Shigri glaciers located in the Chandra basin lying in the western Indian Himalaya were explored using remote sensing data to examine the role of topography in controlling the responses of glaciers towards climatic variations^15^. It was found that the Chhota Shigri can be considered as a representative glacier in the region for reflecting the effect of climatic variability on the glacier response as several glaciers in the region show close similarity with the Chhota Shigri glacier in terms of topographic and climatic settings and morphological characteristics. A review on Himalayan glaciers shows that most Himalayan glaciers are retreating and the average yearly shrinking was estimated to be approximately 17.5 m a^−1^ during the period from 1971 to 2004^2^. The study^16^ on Gangotri glacier shows that its average velocity is 4–6 cm/day corresponding to the yearly shrinking of 15–20 m a^−1^. The geomorphological study^17^ of the Alaknanda glacier in the Ganga basin shows that the Alaknanda glacier accounts 16.77% glaciated region of the Ganga basin and the glaciers in the Alaknanda sub-basin are dirty due to higher percentage of ablation area under debris cover.

It is evident from the literature review that there is lack of mass balance data of glaciers. In fact, not only glaciers in the Himalayan region, but a number of glacierized regions of the world are still unsampled or poorly sampled and inadequately monitored. If higher number of years of records is taken as basis and especially if seasonal mass balances in terms of winter and summer mass balances are required to understand the prevailing glaciological processes, the mass balances of a very few glaciers are available^18^. Currently, there is only one literature^3^ pertaining to the glacier retreat study on the Parvati glacier. This study is based on very limited, short and sparse data set. It has reported mass balance for only one year. There is no gauging site for continuous measurement of meteorological and hydrological parameters. The glacier retreat may have greater ramifications on water, food and energy security. The glaciological processes and the dynamics of glaciers and snow mass are also sensitive to climate change as small fluctuations can sometimes lead to rapid retreat or surge in the glacial extent which may cause devastating flood, glacier lake outburst flood and snow avalanche that may affect life, property and infrastructure adversely on the downstream^19,20^. Further, the increasing mass loss and declining glacier size in mountainous areas and other regions of the earth may contribute significantly to the sea level rise^21^. Thus, an accurate assessment of mass loss and the snow cover helps in understanding the sensitivity of the region. The mass loss and the change in the length of glacier depend upon its geometry and climatic variations^22^. It is therefore essential to have better understanding of glaciers geomorphology and precise assessment of glaciers mass loss. Keeping these points in view, the main objective of this paper is to present a modeling framework to improve the understanding of accumulation and ablation processes taking place in a glacier system and to quantify the mass balance of a glacier using multitemporal multispectral satellite imageries. This modeling framework is advantageous over the currently used approaches as field measurements of snow and meteorological parameters as well as information derived from the subglacial materials are limited and don’t cover the entire glaciated region. Further, getting long time series of such measurements is another challenge. The developed modeling framework has been applied to the Parvati glacier in the western Himalaya to examine the prevailing glaciological processes and to estimate the surface mass loss of the Parvati glacier. This will not only help in better understanding of glaciers, but also in hydrological and avalanche dynamics studies. The study will be also useful in determining the responses of glaciers to global warming and climatic changes, and in identifying the plausible reasons that are responsible for causing aberration in the glacier response as it may be affected by the presence of debris^23^ and the human behaviour^24^.

### Modeling framework

The mass balance reflects the hydrodynamics of glaciers at multiple spatial and temporal scales which elucidates the perspectives on glacier interactions with hydrology, climatology and environment in the region. The glacier response to climatic variations is commonly expressed in terms of surface mass balance of the glacier system. It is expressed as a sum of accumulation and ablation taking place in a glacier system over a specified period of time. The accumulation embraces all kinds of processes through which ice and snow are added to a glacier system, whereas the ablation embraces those processes through which ice and snow are lost from the glacier system. The accumulation processes that are responsible to increase the mass of the glacier include precipitation of snow and ice directly over the glacier surface, refreezing of liquid water, condensation of ice from water vapor and the transport of snow or ice to the glacier system by the kinematics of avalanches or wind. The ablation processes that are responsible to reduce the mass of the glacier include melting, evaporation, wind erosion, calving, and removal of snow and ice from the glacier system by the actions of avalanches. The ice and snow removed by the action of wind from the glacier is termed as ablation due to wind erosion. Mathematically, the mass balance of a glacier can be expressed as an algebraic sum of accumulation and ablation^25,26^:

1 $${m}_{b}={\int }_{{t}_{1}}^{{t}_{2}}\left(c+b\right)dt$$

where m_b_ is the mass balance expressed as the net mass flux over time interval of t_1_ and t_2_, c is the accumulation defined as the rate of gain in the glacier mass, and b is the ablation defined as the rate of loss in the glacier mass. The accumulation is taken as positive, whereas the ablation is considered as negative while computing the mass balance of a glacier.

The annual accumulation (a_c_) and the annual ablation (a_b_) in a year can be expressed as:

2 $${a}_{c}={\int }_{{t}_{1}}^{{t}_{2}}cdt$$

3 $${a}_{b}={\int }_{{t}_{1}}^{{t}_{2}}bdt$$

where t_1_ and t_2_ denote the beginning and ending times of a year for which mass balance is being calculated.

The annual mass balance (m_ba_) is the difference between the annual accumulation and the annual ablation:

4 $$m_{ba} = a_{c} - a_{b}$$

The mass balance of glacier undergoes an annual cycle of growth and decay because the accumulation and ablation of glaciers are seasonally governed. Thus, the annual mass balance of glacier at the end of a balanced year can be also expressed as the sum of the winter mass balance (m_bw_) and the summer mass balance (m_bs_):

5 $$m_{ba} = m_{bw} + m_{bs}$$

To calculate the winter mass balance, t_1_ and t_2_ in Eq. (1) are taken as the beginning and ending of winter season. If the same time limits are used in Eqs. (2) and (3), it will yield the winter accumulation and the winter ablation, respectively. Similarly, if t_1_ and t_2_ are the beginning and ending of summer season, then Eq. (1) will give the summer mass balance and Eqs. (2) and (3) will yield the summer accumulation and the summer ablation, respectively. The winter mass balance is generally positive, whereas the summer mass balance is negative.

The values of integral of a_c_ and a_b_ over the horizontally projected area (S) of the glacier are called total annual accumulation (Ta_c_) and total annual ablation (Ta_b_), respectively:

6 $$T{a}_{c}=\int \int {a}_{c}dxdy$$

7 $$T{a}_{b}=\int \int {a}_{b}dxdy$$

The difference between the total annual accumulation and the total annual ablation is called the total annual net balance or net budget total (A_n_) and the specific net balance or the mean specific budget (A_v_) is calculated by averaging it over the area of the glacier:

8 $$A_{n} = T_{{a_{c} }} - T_{{a_{b} }}$$

9 $$A_{v} = A_{n} /S$$

The annual mass balance of a glacier is generally calculated for a fixed period of one year time interval. It is generally taken as a hydrological year for appraising the glaciers feedback to hydrological systems and predicting the glaciers health under changing climate. The yearly mass loss is a hydrological budget which measures the difference between the accumulated and the ablated glacier masses annually^27^. The difference between the accumulated and the ablated glacier mass is also termed as the net budget or the net balance, which depends upon the minimum mass of snow and ice at the end of each summer. This budget or net mass flux is an important link between the climatic environment and the glacier system which describes how the glacier adjusts dynamically to environmental perturbations, and also between the glacier hydrodynamics and the response of the hydrological system.

A number of studies carried worldwide have shown the potential use of remote sensing techniques in monitoring snow, ice and glaciers using optical and microwave sensors^4,15–17,28–32^. The snow layers bring variation in snow properties spatially in all directions, however, the physical and dielectric properties vary significantly along the vertical drop of the snow layer because of the consolidation of various layers with time^19,28^. The increased availability of digital data from various remote sensing satellites at appropriate spatial and temporal resolution provides capability of regular monitoring of changes in the glacier geomorphology and its parameters over the large areas of interest and the longer time spans required for sustainability analysis. A number of glaciological parameters and properties, namely, snow and ice extent, snout or terminus position, glacier area, glacier length and width, surface elevation, glacier volume, surface flow fields, accumulation and ablation zones, snowline, equilibrium line altitude (ELA), and accumulation area ratio (AAR) could be obtained from the multispectral remote sensing data. These quantities are very useful in understanding the glaciological processes and the glacier mass balance studies. There are direct and indirect methods for the assessment of glaciers condition and determining the glacier mass loss. The mass loss is glacier specific and depends upon the terrain and climatic conditions. The glacier mass loss and snowmelt simulation play very important role in the hydrological models. In this paper, a satellite imagery based model is presented to estimate the glacier mass balance and examine its annual variability. The model combines remote sensing and statistical techniques to compute the glacier mass balance. The mass balance is calculated using a regression equation developed between AAR and in-situ measurements of mass balance. This regression model is based on the hypothesis that a strong relationship exists between the net mass balance and the accumulation area ratio as evident from various literature^33–38^ and the statistical test carried out in the present study. The remote sensing provides a powerful tool to identify the ELA and the transient snowline (TSL) from the multispectral satellite images, and is very advantageous in those areas where field observations are non-existent or lacking^39^. The ELA is defined as the spatially averaged elevation of the equilibrium line, which is an imaginary line joining a set of points on the glacier surface at which the annual net mass balance is zero. The TSL is the locus of locations of the transition from the snow cover to the bare glacier ice, superimposed ice or firn at a specific time during the ablation season. The position of the ELA is obtained by monitoring the position of transient snowlines throughout the ablation season because the altitude of the snowline at the end of the ablation season is considered as the ELA. To examine the snowline, we need to know the altitudinal distribution of snowmelt and accumulation during the mass balance year in order to run the model.

The relation between the mass balance and the accumulation area ratio can be expressed as:

10 $${\text{B}}_{{\text{n}}} {\text{ = a * AAR + b}}$$

11 $${\text{AAR = Accumulation Area / Total glacier Area}}$$

Where ‘a’ and ‘b’ are constants, and B_n_ is the net mass balance of a glacier at a regional scale expressed in water equivalent.

The accumulation area and the total glacier area required in the proposed modeling framework for glacier mass balance computation are determined from the satellite imageries. The snowline can be also mapped using satellite imageries. For mapping the glacier zone and its morphological computation at various times, the image processing is carried out with multi-dates multispectral satellite images using a spatial model for the NDSI (Normalized Difference Snow Index) computation. The ERDAS Imagine and ArcGIS software were used for image processing, morphological computation and spatial mapping. The remote sensing data utilized in the present study includes LANDSAT and MODIS satellite images. For Landsat 5 and 7 ETM + satellite images, the NDSI is computed using reflectance data for band 2 and band 5. Mathematically, it can be expressed as:

12 $$NDSI= \frac{Band 2-Band 5}{Band 2+Band 5}$$

For Landsat 8 (OLS and TIRS) satellite images, the NDSI is computed using reflectance data for band 3 and band 6:

13 $$NDSI= \frac{Band 3-Band 6}{Band 3+Band 6}$$

**Figure 1:**
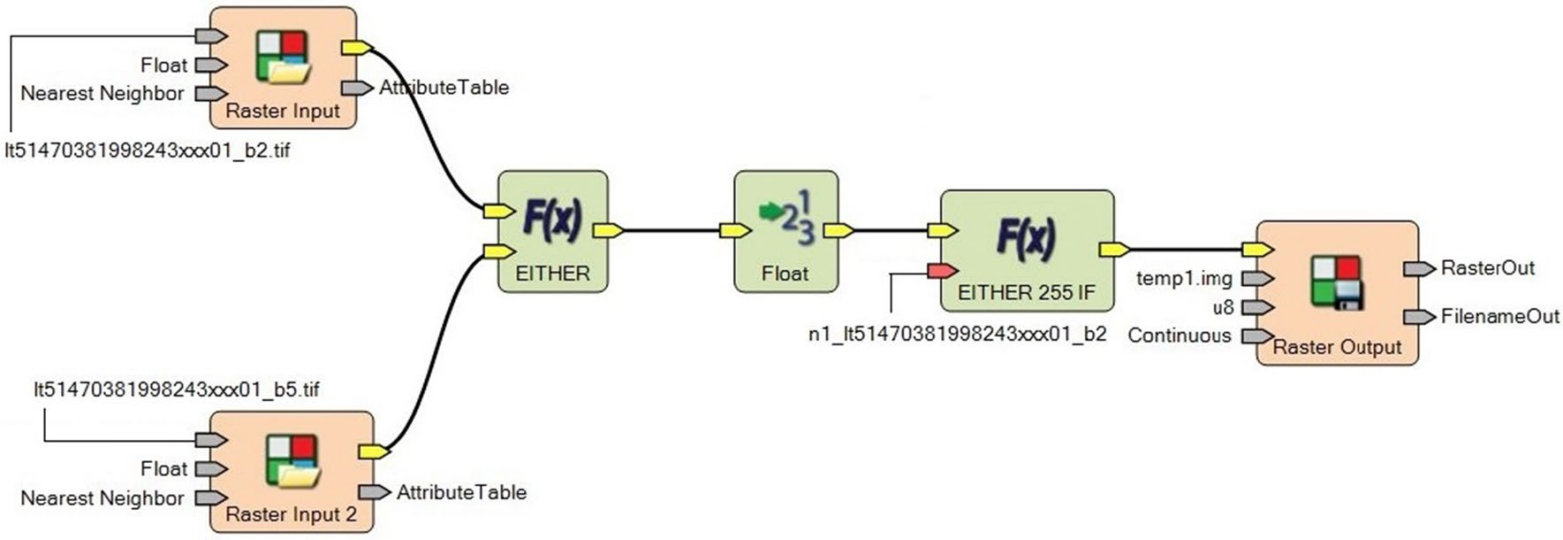
Spatial model for NDSI computation using multispectral satellite images.

The spectral and spatial resolutions of satellite images of various bands used in the NDSI computation are given in Table 1. The spatial model used for the computation of NDSI using satellite images is shown in Fig. 1. For the MODIS satellite images, the present study utilizes MOD10A2 data products of MODIS and is referred as the MODIS/Terra Snow Cover 8-Day L3 Global 500 m Grid (MOD10A2) data sets. It contains data fields for the maximum snow cover extent over an eight-day compositing period and a chronology of snow occurrence observations in compressed Hierarchical Data Format (HDF). MOD10A2 data products consist of 1,200 km by 1,200 km tiles of 500 m resolution data gridded in a sinusoidal map projection. The snow cover data is based on a snow mapping algorithm that employs NDSI to identify the snow covered and no-snow lands. The NDSI is calculated using bands 4 and 6 reflectance data of MODIS.

**Table 1:** Spectral and spatial resolutions of Landsat images used in NDSI computation.

Bands	Wavelength (micrometers)	Resolution (meters)
**Landsat 5 ThematicMapper (TM) & Landsat 7 Thematic Mapper Plus (ETM +)**		
Band 2–Green	0.52–0.60	30
Band 5–Shortwave Infrared (SWIR) 1	1.55–1.75	30
**Landsat 8 Operational Land Imager (OLI) & Thermal Infrared Sensor (TIRS)**		
Band 3–Green	0.533–0.590	30
Band 6–Shortwave Infrared (SWIR) 1	1.566–1.651	30

The accumulation area varies from year to year depending up on the position of the equilibrium line altitude. The altitude of the snowline at the end of the ablation period is taken as the equilibrium line altitude. The ablation period depends upon the climatic season of the study area. In the present study, the month of September (10th September) has been taken as the end of the ablation period for the study area under consideration. Thus, the snow lines have been demarcated and the accumulation areas have been delineated on the satellite images for the years from 1998 to 2016. After delineating the accumulation areas, the spread of the accumulation area has been mapped and the area computation has been performed using GIS for each of the year during the period 1998–2016. The altitudes of snowlines have been estimated using the hypsography derived from Digital Elevation Model (DEM) generated from Cartosat-1 satellite data^40^ and multi-temporal satellite imageries. Cartosat-1 is an Indian remote sensing satellite and the generated DEM is having 30 m resolution. The modeling framework presented in this study also facilitates customization of the estimation of mass balance from other methods, namely, geodetic and statistical based on AAR in an open source image processing tool. The volumetric glacier mass balance can be computed using the following expression:

14 $${\text{Volumetric change }}\left( {\Delta {\text{V}}} \right) \, = {\text{ Mass Balance }}\left( {{\text{MB}}} \right) \, *{\text{ Glacier area }}\left( {\text{A}} \right)$$

The formation of calculating mass balance has been regressed and is based on the published data^41–43^. The glacier mass balance can be estimated by using in-situ field measurements, hydrological balance, statistical technique, or a combination of these methods. The glaciological and geodetic methods are based on in-situ measurements. The glaciological method measures accumulation and ablation in situ by using stakes and pits at representative points on the glacier surface over the entire glacier during a balance year, whereas the geodetic method creates digital elevation models of glacier at different times and computes mass balance by determining changes in the glacier thickness. The hydrological method uses a water balance equation to compute glacier mass balance using snow and meteorological measurements and discharge data. Since these methods require extensive frequent field monitoring, measurements, survey, and/or extensive network of automatic weather stations and well established gauging sites, the use of these methods was not preferred for the study of mass balance of Parvati glacier located in the western Himalaya as the Parvati river basin is poorly monitored and gauged, and very limited literature is available about this river basin, particularly Parvati glacier. The statistical technique attempts to establish relationship between in-situ equilibrium line altitude or accumulation area ratio and specific mass balance^34,39,41,44–46^. The present study utilizes an integrated approach combining in-situ measurements, statistical technique and remote sensing technique. Since the mass balance depends upon the accumulation and ablation over a period of time, the hypothesis of having a constitutive relationship between the mass balance and the accumulation area ratio on the annual basis has been conceptualized. The regression analysis has been performed between the data sets of the accumulation area ratio and the in-situ measured mass balance published by various researchers^41–43^ for the adjoining river basin, namely Chandra basin, which falls in the same western Himalayan region and is climatologically and hydrologically similar to the Parvati basin. The Parvati river basin extends between 31° 45ʹ N to 32° 15ʹ N latitude and 77° 5ʹ E to 77° 50ʹ E longitude, whereas the Chandra basin extends between 32° 5ʹ N to 32° 50ʹ N latitude and 76° 50ʹ E to 77° 50ʹ E longitude.

Further, null hypothesis testing was performed for the regression analysis to investigate the significance of formulated relation between the specific mass balance and the accumulation area ratio in order to ensure the reliability of obtained results for the Parvati glacier. The Microsoft Office Excel was used to perform the linear regression and to compute constants ‘a’ and ‘b’ used in the regression equation. The null hypothesis testing was also performed using the same software through t-test. To further ensure the significance of model formulation and reliability of numerical results, computational runs were also taken using R software as a formal check to support the statistical outcomes for the formulated relation using linear regression.

### Study area

Parvati glacier lies in the western Himalaya and is located in the Kullu district of Himachal Pradesh, a State of northern India. It is spread over a very large area within the Parvati river basin characterized by several mountain ranges and gorges. Parvati river is one of the major tributaries of the Beas river which runs through several districts of Himachal Pradesh and enters to the adjoining State of Punjab. Parvati river joins the Beas river at Bhuntar which is about 10 km away from the Kullu district center in the south direction. It emerges from the Mantalai glacier in the northerly direction and flows in the northwest direction up to Manikaran, a pilgrimage centre for Hindus and Sikhs. The river takes turn from this township and starts flowing in the southwest direction and gradually changes its course in the south direction to join the Beas river at Bhuntar. The river valley connects the adjoining district of Lahaul and Spiti through Sara Umga Pass, Pin Parvati Pass and Debsa Pass, which has a number of other glaciers. A number of geothermal springs and waterfalls are found on the banks of the Parvati river. It has a large potential of hydroelectric projects of varying sizes.

A number of tributaries join the Parvati river in which Basuki Nal and Dibibokri Nal are the two important tributaries. The Basuki Nal river joins the Parvati river near Tunda Bhuj at an altitude of 3,285 m, whereas the Dibibokri Nal river joins the Parvati river at Thakur Kuan having an elevation of 3,560 m from the mean sea level. The Dibibokri glacier and the Dibibokri Pyramid mountain peak having an altitude of 6,400 m are in the upwards towards the northeast. The Mantalai lake is located near Pandupul at an elevation of 4,100 m above the mean sea level and is a source of water for the Parvati river. Pandupul is further up from the Thakur Kuan and is characterized by the natural rock bridges crossing the river valley. The Mantalai glacier is located beyond it and has an elevation of 5,200 m from the mean sea level. The river basin is characterized by high mountainous ranges, steep gorges, rocky outcrops, coniferous forest and alpine flowers.

This river is fed by nearly 299 glaciers covering a total area of 425.318 km^2^. The snow starts melting and turns into snowmelt runoff as we go down from the glaciated region. The average minimum temperature of the region is − 2 °C, however it goes to − 10 °C due to wind factor. From the Mantalai lake, it is possible to see the head of the Parvati river. It has huge snowfield, flanked on both sides by various mountain peaks ranging 5,000–6,000 m.

### Sampling and analysis

The boundary of the Parvati river basin, in which the Parvati glacier lies, was delineated from the topographic map of 1:50,000 scale, which was prepared by the Survey of India in 1962. The geo-referenced catchment boundary was superimposed on the geo-referenced satellite images of 1998–2016 containing Parvati region obtained using Landsat and MODIS satellite images datasets^47,48^. The present study utilized data sets of imageries of these two different remote sensing satellites; satellite images for the period 1998 to 2002 and 2008–2011 from Landsat 5 & 7, and for the period 2013 to 2016 from Landsat 8, and for the period 2003 to 2007 and 2012 from MODIS images^48^. The Landsat series satellite images were obtained from the Earthdata search website of NASA (https://search.earthdata.nasa.gov/) and the MODIS images from the NSIDC website (https://nsidc.org/data/MOD10A2/versions/6), available free for downloads. The satellite images considered for the analysis were of September month because the snow cover is found to be the minimum in this month, and thus the glaciers would be clearly visible as the soil and terrain outcrops in the region would be fully exposed during this month. The delineation of the glacial boundary in the Parvati river basin was carried out using the standard band combination of 2, 3 and 4, whereas the debris cover on the glacier was estimated using band combination of 2, 4 and 5. The wavelength ranges of spectral bands 2, 3, 4 and 5 are 0.52–0.59 μm, 0.62–0.68 μm, 0.77–0.86 μm, and 1.55–1.75 μm, respectively. The reflectance for the debris is much more than that of ice in band 5, which helps in identifying the pixels of debris cover on the satellite image, because the pixels of debris cover appear as red tone on the glacier region when color composite map is generated with said combination of spectral bands. The changes in the glacial parameters were analyzed using a Geographic Information System (GIS), which is a powerful tool for storing, retrieving, analyzing and visualizing spatial and non-spatial multidimensional data.

The procedure to obtain the glacier boundary and the glacier accumulation area from satellite images involves a series of steps involving image interpretation, image processing, digitization, and map analysis techniques. A false color composite (FCC) map is made using reflectance values of spectral bands of 2, 3 and 4 of Landsat images. The image interpretation keys, namely, tone, texture, pattern, shape, size, site, shadow, and association are used to distinguish various features from the FCC of satellite image. The image processing was carried out using ERDAS Imagine software. The pixel wise reflectance data of spectral bands of 2, 3 and 4 of Landsat images were assigned, respectively, to Blue, Green and Red colors to make the FCC. This is the common method practiced worldwide for making the standard combination of spectral bands to generate FCC maps from remote sensing satellite images. The glacier area becomes clearly visible on the satellite image having distinct tonal values from the non-glacier areas. The glacier areas appear as sky blue or aqua color in FCC. Based on this tonal difference, the boundaries are delineated and digitized online to obtain the boundary of glacier extent. Sometimes, debris may be appearing on the glacier surface depending upon the climatic and terrain characteristics. The band 5 reflectance for rock or debris cover is substantially higher than that of ice. The wavelength of band 5 falls in the Shortwave Infra Red (SWIR) zone of electromagnetic spectrum. Thus, the debris cover appears as red tone when a color composite map is generated using band combination of 2, 4 and 5, which is very helpful in delineating the debris cover areas. The accumulation and the ablation depend upon the climatic season of the study area. The accumulation takes place during the winter season, December to March; whereas the ablation takes place during the summer period, March to July. However, the melting may continue till the end of the monsoon period, July–September. Thus, September is considered as the end of the ablation period as the snow cover is the minimum and the glacier is fully exposed. Similarly, the accumulation area can be interpreted from the satellite images of February month end or March beginning when the snow cover is the maximum and the accumulation zones of glacier would be clearly visible. The accumulation area is the area of the glacier above the equilibrium line. The snow lines are demarcated and the altitudes of snowlines are calculated using the hypsography derived from 30 m resolution digital elevation model. The accumulation area is obtained by calculating the glacier areas above the equilibrium line from the altitudinal variation of satellite derived glacier and snow cover areas.

Identifying the glacier boundary by making use of standard combination of bands 2, 3 and 4 is based on the FCC where a composite map is generated based on the spectral reflectance values. FCC is generally used for identifying the different features present in a raster image. The NDSI is different from the FCC, as the NDSI is obtained by image processing following a raster based mathematical calculation from the spectral reflectance values of bands 2 and 5 for Landsat 5 and 7 imageries, bands 3 and 6 for Landsat 8 imageries, and bands 4 and 6 for MODIS imageries. The NDSI method uses band addition, band subtraction and band ratio techniques for the computation of NDSI values from the spectral reflectance data of the specified bands. It identifies the snow-ice pixels in the raster image of the basin and is used for the snow and glacier mapping.

The snow and glacier boundaries are distinguished based on the tonal values that represent the relative differences in the spectral reflectances. The snow having relatively lesser density and more wetness show relatively less reflectance as compared to the glacier in the green band of optical region. This relative difference is also observed in NDSI. The pixels of glacier area show the highest DN values (255) in raster maps. The satellite data utilized in the present study were selected for those days that were having cloud free data. However, there are algorithms available in literature^49–51^ to distinguish snow or ice and cloud, and for the cloud removal and rectification of the satellite data due to cloud effect. These algorithms are based on the differences between reflectance values of cloud and snow or ice and their emittance characteristics. The clouds are highly variable and show high reflectance in the visible and Near Infra Red (NIR) region, whereas the snow or ice shows low reflectance in the Shortwave Infrared (SWIR) region of the electromagnetic spectrum.

As described above, data sets from two different satellites were collected and analyzed. These satellites data are of different spatial resolution. In the present study, MODIS data was used for the years 2002–2007 because the snow cover area was missing in the Landsat data for these years. Therefore, this data was merged to improve the interpretation capability. This technique was found very suitable for the glaciated region. In order to verify the results of the present study, glaciers delineated in the satellite images are compared with its relative positions and geomorphological features reported by past studies published in various literature including the collection of imageries and widespread of the world’s glaciers captured in aerial photography and maps^52–55^. Inventory data of several glaciers worldwide have been compiled and documented by UNESCO and World Glacier Monitoring Service^37,52^. The glacier inventory data for Indian Himalayan glaciers is documented by Geological Survey of India^56^. A number of literature^52–55^ are available that deal with the instructions and guidelines for the compilation and assemblage of data for glacier inventory. In the present study, the glaciers delineated in the satellite imageries are verified by its relative position and geomorphological features that include coordinates, orientation, maximum length and mean width. Further, since the spatial resolutions of MODIS and Landsat images are different, mapping of snow and glacier cover was carried out with both types of satellite images for the period for which both satellite images were available, and comparative analysis was carried out to ensure the consistency of calculation results. It was found that the average error associated with the MODIS images for the calculation of snow and glacier covered areas due to difference in spatial resolution is 2%, which is within the acceptable accuracy as reported in literature^57,58^.

## Results and discussion

**Figure 2:**
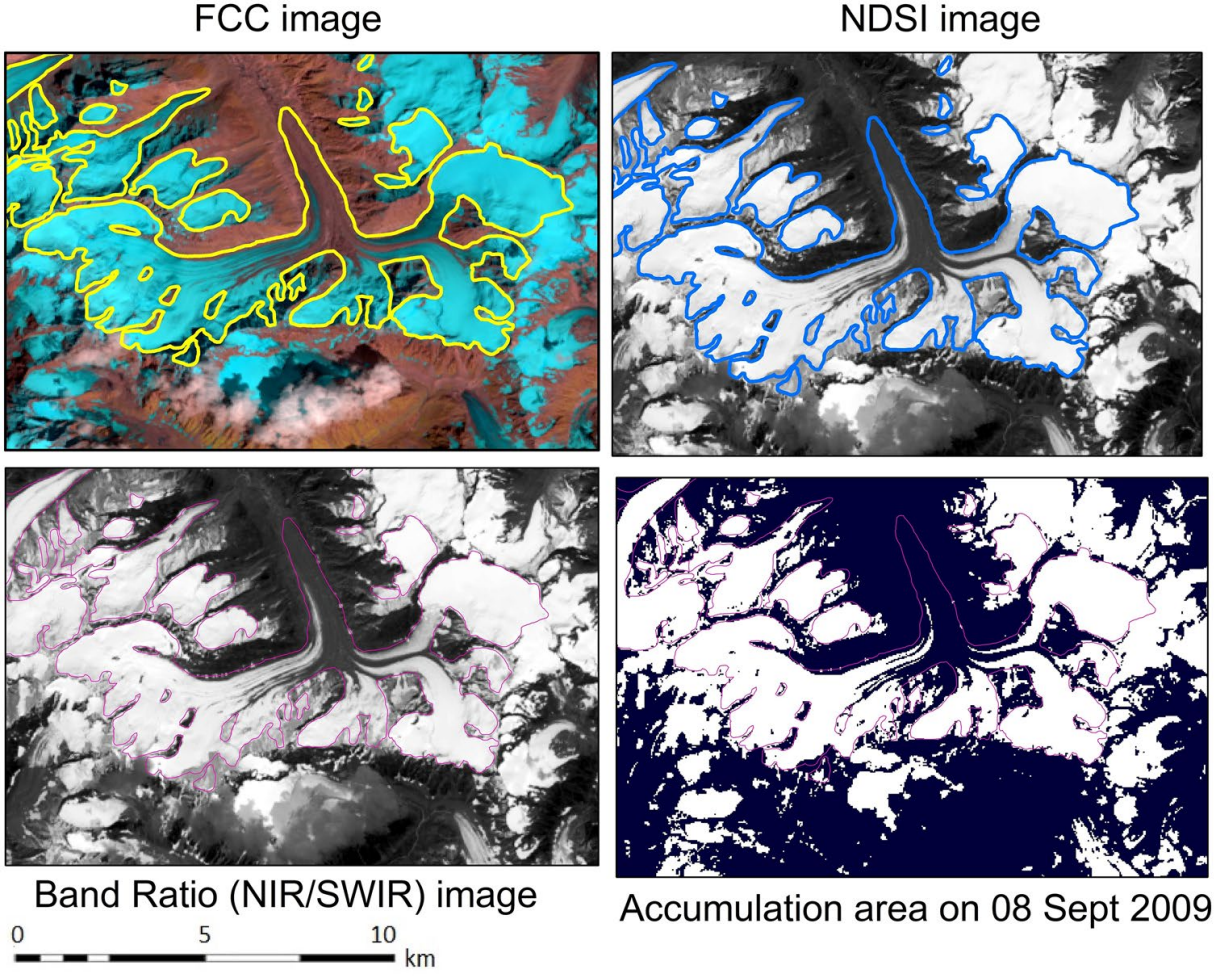
FCC, NDSI, band ratio and accumulation area obtained from satellite images for a part of Parvati glacier by using ERDAS IMAGINE 14.0 (https://www.hexagongeospatial.com/) and ArcGIS 9.3 software (https://www.esri.com/).

The proposed modeling framework was applied to the Parvati glacier for investigating the mass loss during the period of 19 years from 1998 to 2016. The sensor and band characteristics of remote sensing data utilized in the present study are given in Table 2. Figure 2 shows the spatial distribution of the accumulation area as obtained on 8^th^ September 2019 for a part of the Parvati glacier. The first image on the left top corner shows the delineation of the glacial extent from the FCC (False Color Composite) image of the area, whereas the second image on the right top corner shows the glacial extent as delineated from the NDSI image. The satellite image processing was also carried out using band ratio technique to delineate the glacial extent as shown in the left bottom corner of this figure. The band ratio of satellite images was carried out pixel by pixel as a raster model using the reflectance values of NIR and SWIR bands. The accumulation area as shown in the right bottom corner image was finally obtained based on these delineated glacial extents derived from satellite images using the FCC, NDSI and band ratio techniques. The first technique involves making a False Color Composite (FCC) from multispectral satellite imageries and identifying the snow-ice pixels that help in demarcating the extent of the glacier and delineating its boundary by online digitization. The NDSI technique involves automatic identification of snow-ice pixels by image processing following a specified mathematical formula for different satellite imageries. The band ratio technique is another image processing technique that involves ratio of two spectral bands. The calculation is carried out pixel by pixel by computing the ratio of reflectances of two specified spectral bands (NIR and SWIR in the present study), and the glacier extent is determined by the spread of pixels of ice and snow through image interpretation as discussed earlier. The FCC technique performs well in delineating the boundary. However, the NDSI technique performs best in delineating the glacier spread and its areal extent. This analysis was carried out for all sized glaciers in the Parvati river basin.

**Table 2:** Specifications of remote sensing data utilized in the mass balance study.

Specification	Data Set 1	Data Set 2	Data Set 3	Data Set 4
Platform	Landsat 5 and 7	Landsat 5 and 7	Landsat 8	MODIS
**Sensors and bands**	Landsat TM and ETM + (bands 3, 4 and 5)	Landsat TM and ETM + (bands 2, 3 and 5)	Pushbroom, The Operational Land Imager (OLI) and Thermal Infrared Sensor (TIRS) (both OLI and TIRS)	MODIS/Terra Snow Cover Daily L3 Global 250 m (bands 1–2) 500 m (bands 3–7) 1,000 m (bands 8–36)
**Ground resolution**	30 m	30 m, 15 m	30 m (visible, NIR, SWIR); 100 m (thermal); and 15 m (panchromatic)	500 m 8 Days MOD10A2 data set
**Precision**	15 m (horizontal)	15 m, 7.5 m	15 m, panchromatic	500 m × 500 m
**Scenes (path and Row)**	147 and 38	147 and 38	147 and 38	146 and 37 146 and 38 147 and 36 147 and 37 147 and 38 148 and 36 148 and 37 148 and 38
**Scenes Year**	1998,2001,2008,2010 2011	1999, 2000, 2002, 2009	2013,2014,2015, 2016	2003,2004,2005,2006, 2007,2012
**Data Type Level-1, Sensor Identifier, UTM Zone, Map Projection Level-1, Datum**	ETM_L1TP, ETM(Landsat_7), TM_L1TP, TM(Landsat_5) 43, UTM, WGS43	ETM_L1TP, ETM(Landsat_7), TM_L1TP, TM(Landsat_5) 43, UTM, WGS43	OLI_TIRS_L1TP, OLI_TIRS (Landsat_8), 43, UTM, WGS43	MODIS Sinusoidal , 43, UTM,WGS43,

Figure 3 shows the spatial distribution of Parvati glacier superimposed on the FCC of the Parvati basin, which was obtained from the multispectral satellite images using the procedure as described earlier. The NDSI image for the whole Parvati basin is shown in Fig. 4. It is evident from Figs. 3, 4 that the snow covered areas and the number of glaciers are more in the northern side of the river as compared to the snow covered areas and the number of glaciers in the southern side of river. The spatial distribution of accumulation area of the glacier in the Parvati river basin is shown in Fig. 5. It is evident from this figure that a significant area, almost more than 50% is the accumulation area. Figure 6 shows the annual variation of accumulation area of Parvati glacier as derived from the multispectral satellite images. It is evident from this figure that the accumulation area varies in between 140 and 300 km^2^, having the minimum value in the year 2002 and the maximum value in the year 2009. It also shows that the average accumulation area as obtained from the 19 years of multispectral satellite images during the period 1998 to 2016 comes out to be 232.86 km^2^. The accumulation area in the Parvati glacier has been always more than 200 km^2^ except the years 2002 and 2006. The annual variation of AAR is shown in Fig. 7. The value of AAR varies between 0.33 and 0.70. It is evident from this figure that the Parvati glacier is not in equilibrium, it is very dynamic and its behavioural response changes year to year. The value of AAR has remained in between 0.5 and 0.6 for most of the years during 1998–2016. This value has become more than 0.6 in years 2004 and 2009, indicating events of positive mass balance, and therefore, increase in the glacier mass in these years. The value of AAR decreases to less than 0.50 in years 2002, 2003 and 2006, indicating high glacier mass loss in these years. However, the average value of AAR for the period 1998–2016 comes out to be 0.55 (less than 0.6), which indicates a negative mass loss for the Parvati glacier. This finding is supported by the results obtained for the temperate alpine glaciers^3,59^.

**Figure 3:**
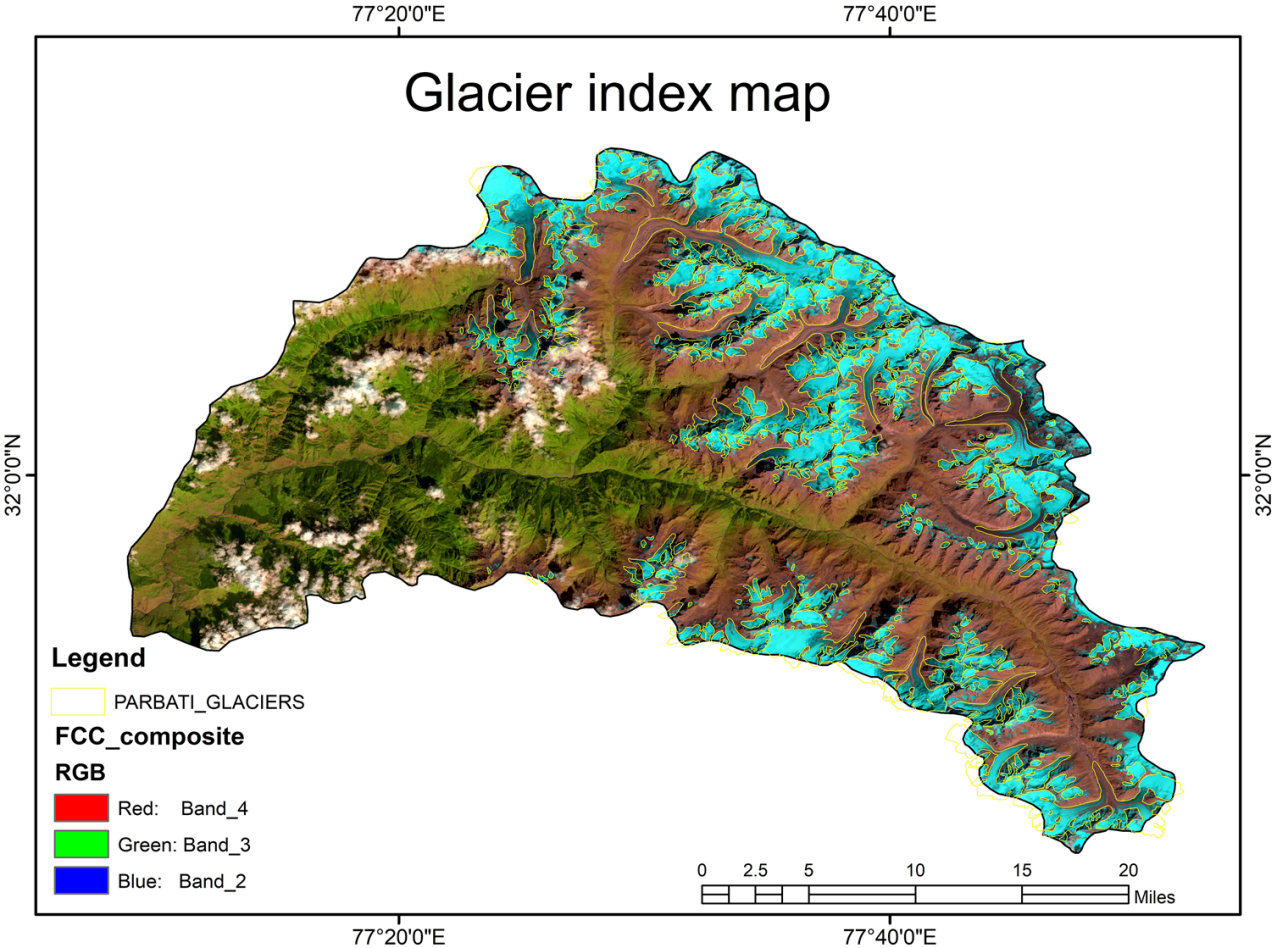
Spatial distribution of Parvati glacier superimposed on the FCC image of Parvati river basin obtained by using ERDAS IMAGINE 14.0 (https://www.hexagongeospatial.com/) and ArcGIS 9.3 software (https://www.esri.com/).

**Figure 4:**
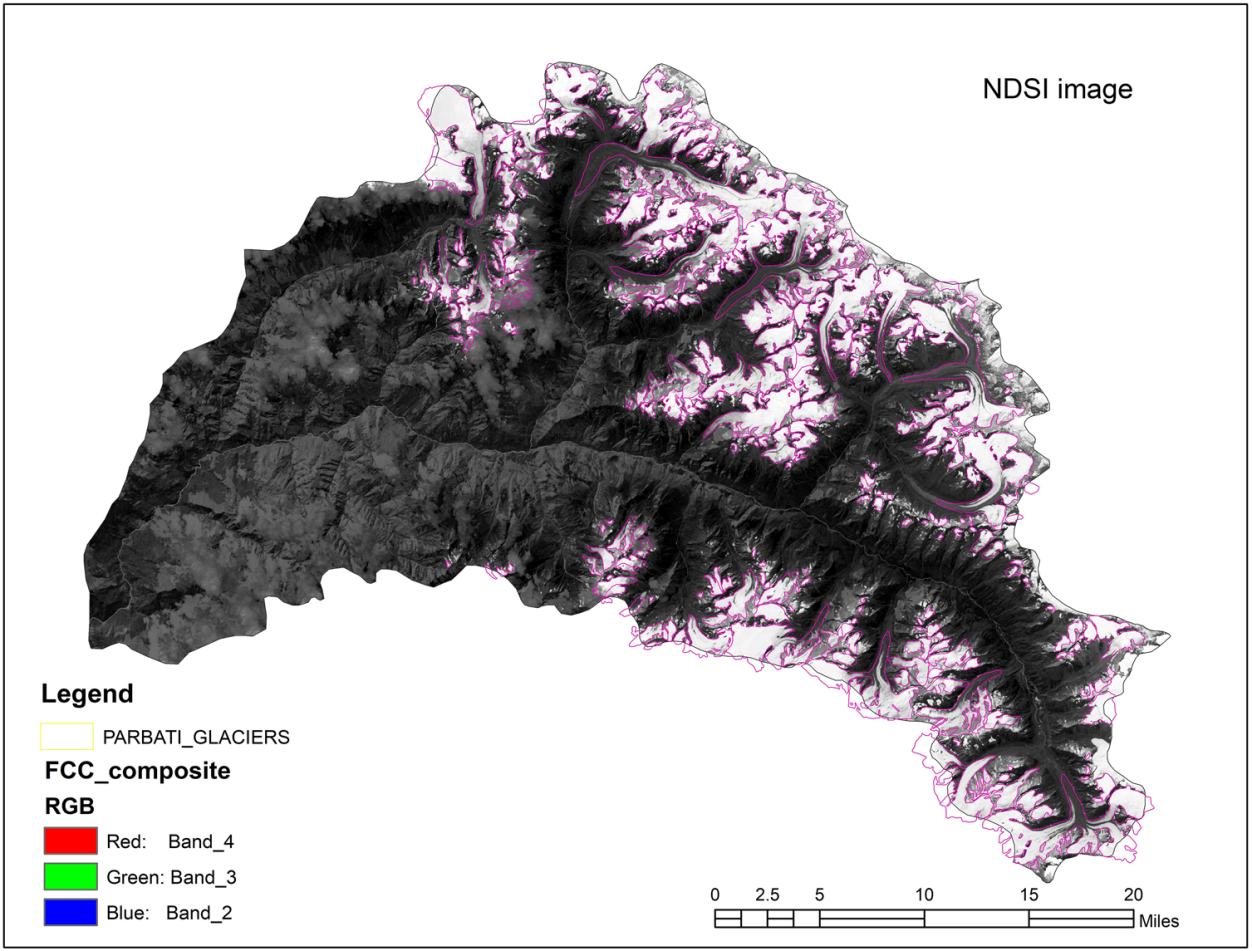
NDSI image derived from multispectral satellite images for Parvati river basin obtained by using ERDAS IMAGINE 14.0 (https://www.hexagongeospatial.com/) and ArcGIS 9.3 software (https://www.esri.com/).

**Figure 5:**
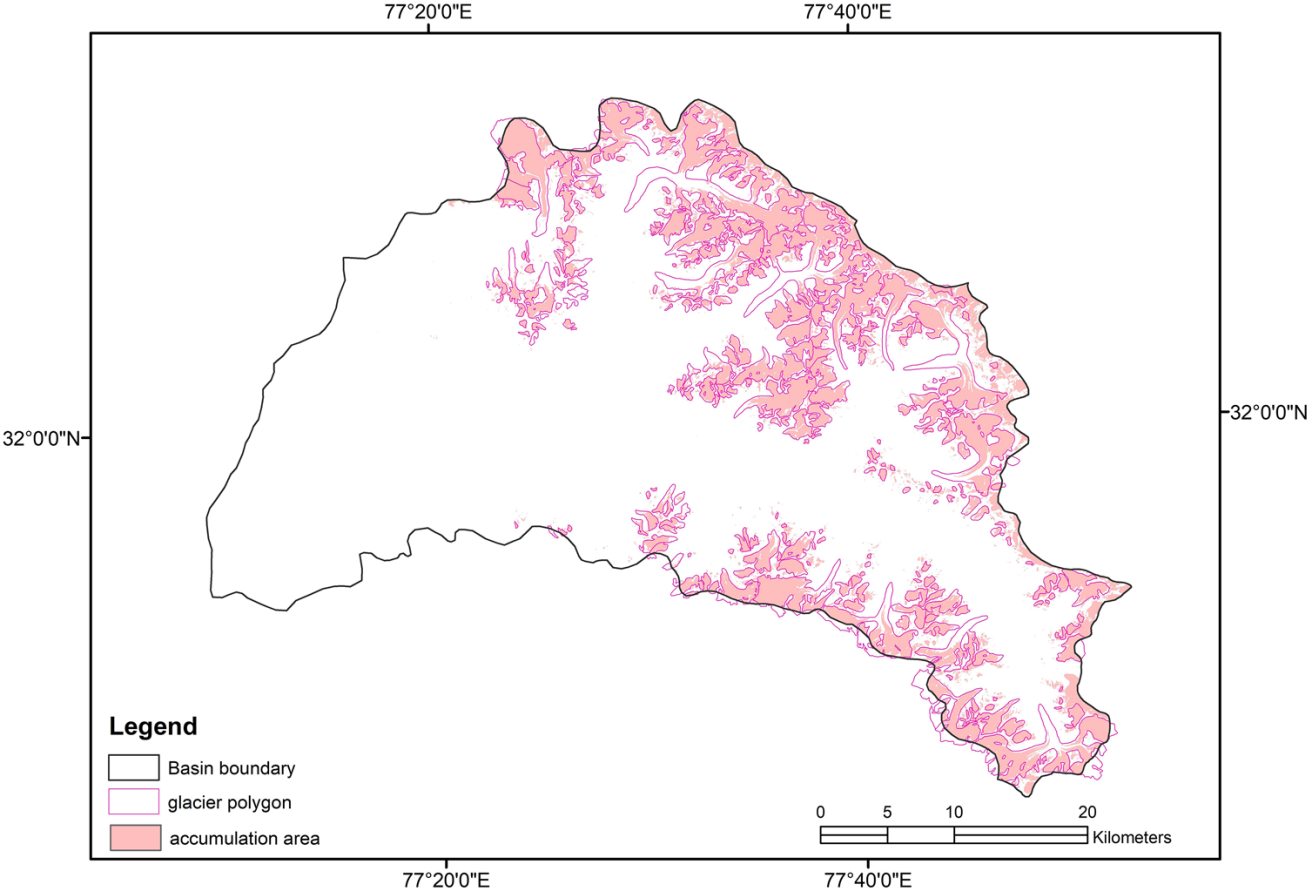
Spatial distribution of accumulation area of glacier in Parvati river basin.

**Figure 6:**
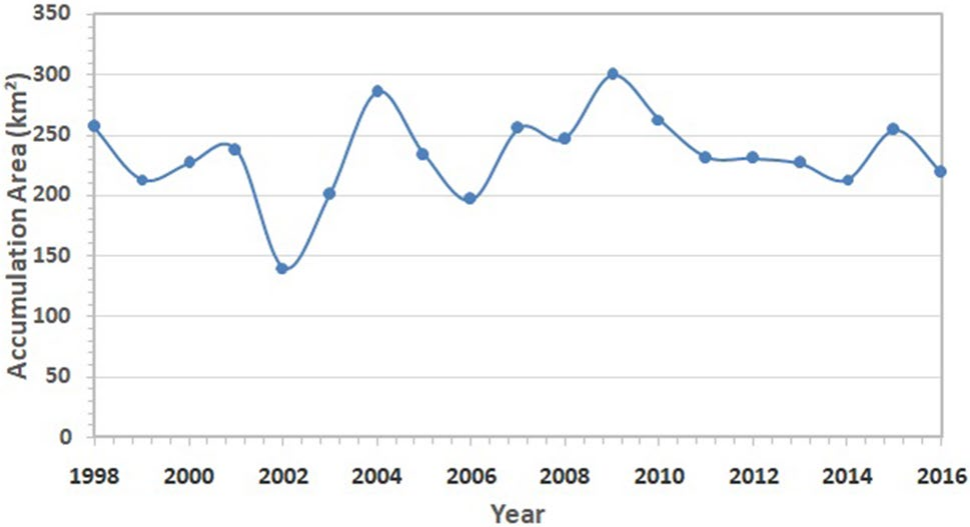
Annual variation of accumulation area of Parvati glacier.

**Figure 7:**
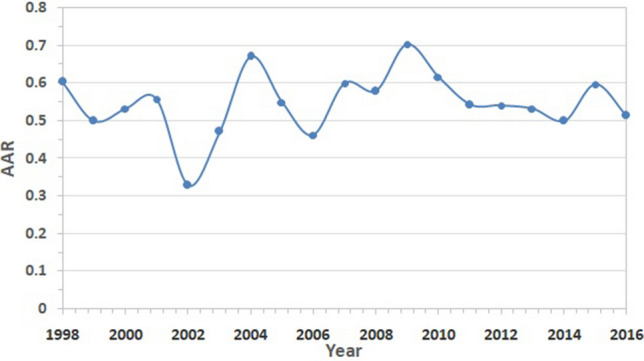
Annual variation of AAR of Parvati glacier.

### Annual variation of mass loss

**Figure 8:**
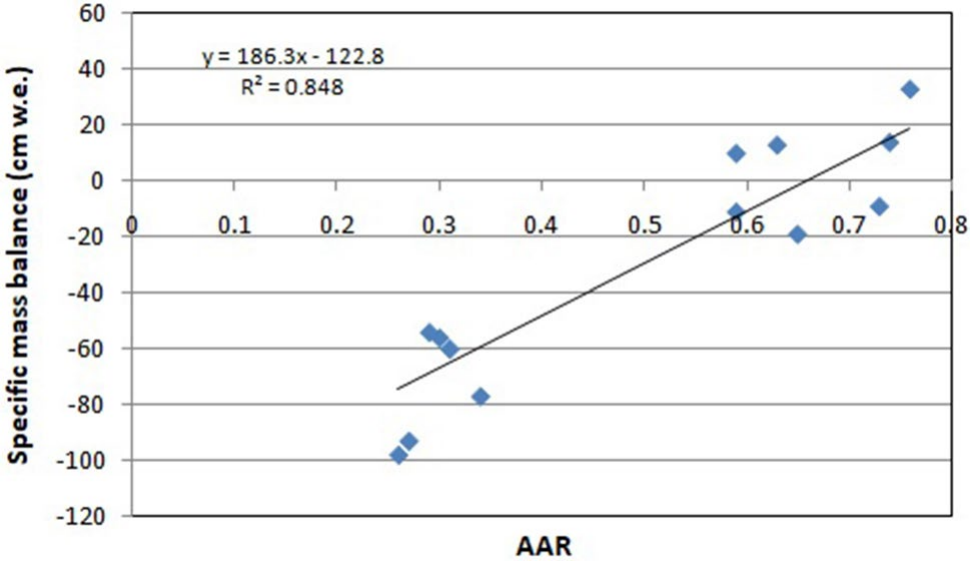
Formulated relationship between specific mass balance and accumulation area ratio as obtained from the linear regression using in situ field measurements and having p-value of 0.011 at 5% significance level.

The mass balance of the Parvati glacier was carried out using the developed satellite imagery based model for the period 1998 to 2016. For computing the mass balance of the Parvati glacier in the present study, the values of constants ‘a’ and ‘b’ in the mathematical model were taken to be equal to 186.3 and − 122.8, respectively. This is based on the regression relationship established between the annual specific mass balance (cm water equivalent) and the accumulation area ratio for the glaciers in the adjoining basin, namely, the Chandra basin with a high correlation coefficient (r^2^ = 0.85) as the mass balance measurements of the Parvati glacier are not available. The values of these constants are derived based on the field measurements for the periods of 1987–89 and 2003–12^41–43^. Figure 8 shows the formulated relationship between the annual specific mass balance and the accumulation area ratio as obtained from the linear regression. It is evident from this figure that the data points are close to the trendline which has a high value of the coefficient of determination (R^2^) expressed as the square of the correlation coefficient (r).

The null hypothesis testing as described in the modeling framework was carried out for the regression analysis to investigate the significance of model formulation involving formulated relation between the specific mass balance and the accumulation area ratio and to ensure the reliability of obtained results for the Parvati glacier. The significance level is 5% (0.05). The computed p-value is 0.011, which is less than 0.05. Since the p-value is strictly less than the significance value, data used is highly significant. Thus, it shows that the data is highly significant and there is strong evidence in favour of the formulated relationship in the developed model. Further, the computed p-values using the Microsoft Office Excel and the R software are 0.011221 and 0.011220, respectively, for the same data configuration and statistical hypothesis. It clearly demonstrates that the results obtained from both the software are in agreement. Thus, the statistical outcomes support the significance of the model formulation and ensure the reliability of obtained numerical results and findings in the present study.

**Figure 9:**
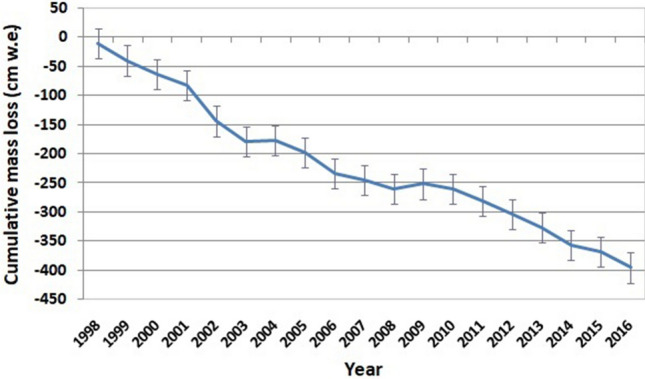
Annual variation of cumulative mass loss of Parvati glacier during 1998–2016.

**Figure 10:**
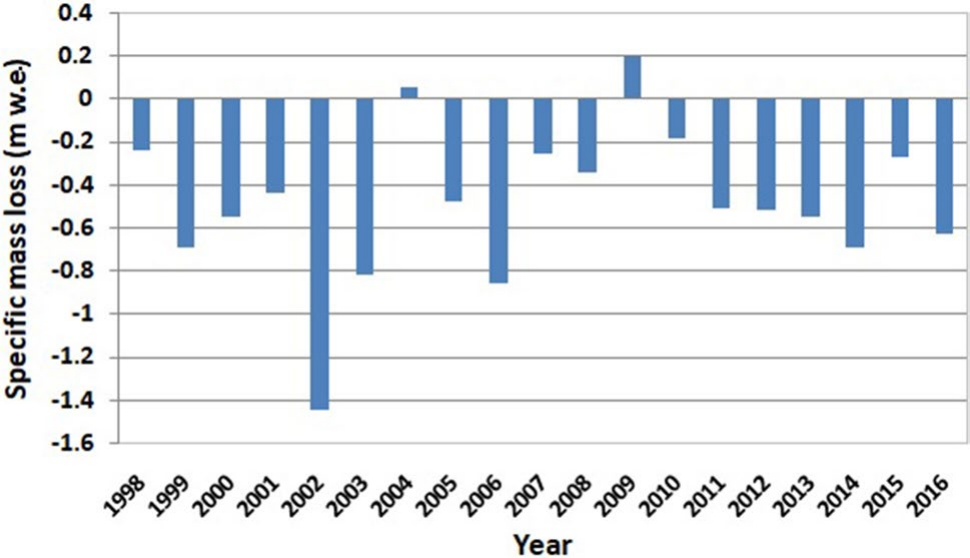
Annual variation of specific mass loss of Parvati glacier during 1998–2016.

In the present study, the mass balance of the Parvati glacier has been computed for a period of 19 years between 1998 and 2016, where systematic remote sensing data is available. The annual variation of cumulative mass loss is shown in Fig. 9. It is evident from this figure that there is an alarming retreat of Parvati glacier with time. Figure 10 shows the annual variation of the specific mass loss of the Parvati glacier. The mean specific mass loss for the Parvati glacier during the period from 1998 to 2016 is found to be equal to − 0.49 ± 0.11 m w.e. It is evident from this figure that there has been significant mass loss of the Parvati glacier in all the years of the study period except the years 2004 and 2009. In the year 2009, Parvati glacier has showed a gain in the mass by 8.26 cm w.e. A small gain in the glacier mass by 2.20 cm w.e. is also observed during the year 2004. The annual glacier mass loss along with error estimates for the period 1998–2016 is shown in Table 3.

**Table 3:** Annual mass loss with error estimates derived from satellite images for Parvati glacier for the period 1998–2016.

Year	Total mass loss at regional scale (cm w.e.)	Error in total mass loss (cm w.e.)	Mean specific mass loss (m w.e.)	Error in mean specific mass loss (m w.e.)
1998	− 10.33308889	1.124669111	− 0.24294972	0.112466911
1999	− 29.60444538	0.931955546	− 0.696054373	0.093195555
2000	− 23.6503689	0.991496311	− 0.556063202	0.099149631
2001	− 18.85574511	1.039442549	− 0.443332874	0.103944255
2002	− 61.51633296	0.61283667	− 1.44636091	0.061283667
2003	− 34.987337	0.87812663	− 0.822615949	0.087812663
2004	2.195745771	1.249957458	0.051625978	0.124995746
2005	− 20.574994	1.02225006	− 0.483755543	0.102225006
2006	− 36.82529072	0.859747093	− 0.865829585	0.085974709
2007	− 11.06415294	1.117358471	− 0.260138366	0.111735847
2008	− 14.83554987	1.079644501	− 0.348810769	0.10796445
2009	8.257138423	1.310571384	0.194140347	0.131057138
2010	− 7.914751786	1.148852482	− 0.186090215	0.114885248
2011	− 21.67049502	1.01129505	− 0.509512765	0.101129505
2012	− 22.01040257	1.007895974	− 0.51750461	0.100789597
2013	− 23.58729327	0.992127067	− 0.554580179	0.099212707
2014	− 29.60619748	0.931938025	− 0.696095568	0.093193803
2015	− 11.59153527	1.112084647	− 0.272538084	0.111208465
2016	− 27.02885723	0.957711428	− 0.63549761	0.095771143

It is evident from these figures that the rate of mass loss is high and alarming. The cumulative mass loss is having almost rapid linear decline rate. The total mass loss from the Parvati glacier during the period 1998–2016 comes out to be equal to 3.95 Gt. The negative mass balance denoting glacier mass loss can be attributed to less snowfall and increased temperature because of global or regional warming and precipitation deficiency in the basin as a result of climate change. It is also observed that the most of the glacier extent is located in the low altitude zone, lower than 5,200 m altitude, and thus it is more exposed to climatic variations. The snowstorm and snowslide may be some other reasons for the mass loss from the glacier^60^. Therefore, it can be inferred that the mass loss in the Parvati glacier is the clear indication of atmospheric conditions including the advance or retreat of the glacial region, which might have resulted due to various climate change aspects^61,62^. This finding is supported by the earlier studies^3,63^, although the first study is based on the limited four satellite datasets up to the year 2001 and the second study has been conducted for a different basin, but is located nearby in the similar climatic zone and same Himalayan range. The analysis of temperature records during the period of 1998–2016 shows that May to September are the months of high temperature and the temperature varies in between 34 to 21 °C, the higher temperature value being in May month. It shows that the melting of glaciers starts in May and the highest mass loss is expected by the end of the September. This observation is also reflected and corroborated in the results obtained for the mass loss of the Parvati glacier as estimated from the satellite imageries. The varying climatic conditions lead to glacier melt which significantly affect the availability of flow in the river. Because of this reason, summer flow increases in some of the river systems for several years and the flow reduces as the glaciers disappear^64^.

A comparison is also made for the results of the specific mass balance obtained for the Parvati glacier with that available in literature for the nearby glaciers falling in the same climatic zone of the western Himalaya and within the same northern State of Himachal Pradesh to verify the consistency and reliability of results obtained in the present study. Results obtained in the present study have been verified for the mass balance by comparing the computed mass balance of Parvati glacier with the measured mass balances of Chhota Shigri, Bara Shigri and Hamtah glaciers located in the Chandra basin which have been published in various literature^65–67^. This comparison has been shown in Table 4. The values of the specific mass balances reported in this table are based on the geodetic method except for the Parvati glacier wherein it has been obtained using the remote sensing method as discussed in this study. Table 4 shows that the computed mean specific mass balance for the Parvati glacier is in good agreement and consistent with the published values of mass balances obtained using the geodetic method for the adjoining glaciers on individual basis as well as on the region-wide. The study reveals that there is good consistency in the mean annual specific mass balances, inter-annual mass changes and inter-annual variability at the regional scale between the glaciers related to homogeneous meteorological conditions during the period 1998 to 2016. Since the Parvati river basin has large number of glaciers (299) of varying sizes, it can be decomposed into homogeneous glaciers following the concept and findings described in literature^12,13^ and to obtain a statistically optimum design of in-situ measurements for mass balance. However, a dedicated field monitoring plan may be required for the measurements of mass balance and snow and meteorological parameters to arrive at the right configuration of decomposition. The findings obtained in this study are consistent with the annual glacier mass changes quantified from observed years at the scale of the Himalayan region and in other regions of the world.

**Table 4:** Comparison of specific mass balance of glaciers in Himachal Pradesh.

Sr. no	Glacier	Basin	Annual specific mass balance (m w.e.)	Method	Time period	Author
1	Chhota Shigri	Chandra	− 0.39	Glaciological	1987–1989	Dobhal et al. (1995)^65^
2	Chhota Shigri	Chandra	− 0.39	Geodetic	1999–2011	Gardelle et al.(2013)^66^ Vincent et al. (2013)^67^
3	Bara Shigri	Chandra	− 0.48	Geodetic	1999–2011	Gardelle et al.(2013)^66^
4	Hamtah	Chandra	− 0.45	Geodetic	1999–2011	Vincent et al. (2013)^67^
5	Region-wide	Lahaul & Spiti region	− 0.44	Geodetic	1999–2011	Vincent et al. (2013)^67^
6	Parvati	Parvati	− 0.49	Remote Sensing	1998–2016	Present study

## Conclusions

The modeling framework developed in this study provides an efficient tool for improved understanding of glacier geomorphology, accumulation and ablation processes and to compute glacier mass balance using multispectral satellite images to understand the hydrodynamics of glaciers and its interaction with hydrology, climatology and environment in the region. It utilizes an integrated approach combining remote sensing, in-situ measurements and statistical technique. The image processing techniques utilized in the study include image interpretation, false color composite, band ratio, and NDSI. The formulated relationship between the mass balance and the accumulation area ratio has a significance of 5% and the null hypothesis testing was performed through t-test to investigate the significance and reliability of results. The developed methodology has been applied to the Parvati glacier in the western Himalaya. The analysis of 19 years of multispectral satellite images of the period from 1998 to 2016 reveals that the snow covered areas and the number of glaciers are more in the northern side of the Parvati river as compared to that in the southern side of river. A significant area of the Parvati glacier, almost more than 50%, is the accumulation area. The accumulation area varies in between 140 and 300 km^2^ with an average value of 232.86 km^2^ and having the minimum value in the year 2002 and the maximum value in the year 2009. The accumulation area in the Parvati glacier has been always more than 200 km^2^ except the years 2002 and 2006. The study shows that the Parvati glacier is not in equilibrium. It is very dynamic and its behavioural response changes year to year. The value of AAR varies between 0.33 and 0.70 during the period 1998–2016, with an average value of 0.55 indicating a negative mass loss. The mean specific mass loss for the Parvati glacier during the period from 1998 to 2016 is found to be equal to -0.49 ± 0.11 m w.e. There has been significant mass loss of the Parvati glacier in all the years except the years 2004 and 2009 showing a gain of mass by 2.20 and 8.26 cm w.e., respectively, in these two years. The computed mass balance of the Parvati glacier is in good agreement and consistent with the values reported for the adjoining glaciers and for the Lahaul and Spiti region of the western Himalaya. The study shows that the rate of mass loss is high and alarming indicating a high retreat of glacier. The cumulative mass loss is having almost rapid linear decline rate. The total mass loss from the Parvati glacier during the period 1998–2016 comes out to be equal to 3.95 Gt. The negative mass balance denoting glacier mass loss can be attributed to less snowfall, increased temperature and snowslide as a result of climate change and higher exposure to climatic variations as the most of the glacier extent is located in the low altitude zone. The mass loss in the Parvati glacier is the clear indication of atmospheric conditions including the advance or retreat of the glacial region, which might have resulted due to factors affecting climate change. It is also observed that the melting of glaciers starts in May and the highest mass loss takes place by the end of the September. The varying climatic conditions lead to glacier melt which significantly affect the availability of flow in the river causing increased summer flow and a probability of drastic reduction in river flow when the glacier disappears.

## Methods

This paper covers a modeling framework to understand accumulation and ablation processes and the estimation of glacier mass balance using multispectral satellite images. The developed modeling framework has been applied to the Parvati glacier in the western Himalaya to estimate the annual glacier mass balance during the period 1998–2016. The pre-processing of satellite data, generation of digital database and mapping glacial extents were carried out using ERDAS Imagine and ARC GIS software. The areal extents of glaciers in the year 2000 were obtained from RGI 5.0 (Randolph glacier inventory). The Landsat series satellite images from the year 1998 to 2016 are obtained from the Earthdata search website of NASA (https://search.earthdata.nasa.gov/), available free for downloads. The scenes at the end of the ablation period (September) were mostly preferred, since the glaciers will be fully exposed and snow cover is at the minimum extent. To enhance the spatial resolution of images, the pan sharpening method (principal component) is applied for the Landsat 8 imagery, whereas for the Landsat 7 ETM + images, the principal component analysis is used.

## Data Availability

To quantify the overall glacier mass loss in the Parvati glacier over the study period, we have used data sets of imageries of two different remote sensing satellites and DEMs; satellite images for the period 1998 to 2002 and 2008–2011 from Landsat 5 & 7, and for the period 2013 to 2016 from Landsat 8 (https://search.earthdata.nasa.gov/), and for the period 2003 to 2007 and 2012 from MODIS images^48^ (https://nsidc.org/data/MOD10A2/versions/6). Particularly, September month was taken for selecting the satellite images in order to capture melting of the glacier which generally ends up by this time. Regarding the detection of the annual end of the summer snowline, we had also used 844 images from different satellites (Landsat 5, 7 & 8 and MODIS) to cover the 299 glaciers for the 19 years study period. From Eq. (1), we can quantify the mass balance of the glacier. It is easily observed that there is good consistency in the inter-annual mass changes between the glaciers related to homogeneous meteorological conditions and the inter-annual variability at the regional scale during the period 1998 to 2016. The glaciers in the Parvati river basin can be decomposed into homogeneous glaciers on the concept published in literature^13^ for the statistically optimum design of in-situ measurements of mass balance. However, more field measurements are required for snow and meteorological parameters to do so and to arrive at the right configuration of decomposition. These results are in favour of consistency as reflected in the inter-annual glacier mass changes quantified from observed years at the scale of the Himalayan region and in other regions of the world. The other aspect is the cumulative mass loss at decadal time scales which varied considerably from one glacier to another. The study also covers the changes in the glacial region and it is noted that the lengths are not representative of changes in mass balance over the period under consideration.
